# Contribution of Glycation and Oxidative Stress to Thyroid Gland Pathology—A Pilot Study

**DOI:** 10.3390/biom11040557

**Published:** 2021-04-10

**Authors:** Aleksandra Kuzan, Emilia Królewicz, Karolina Nowakowska, Kamilla Stach, Krzysztof Kaliszewski, Paweł Domosławski, Łukasz Kotyra, Andrzej Gamian, Irena Kustrzeba-Wójcicka

**Affiliations:** 1 Department of Medical Biochemistry, Wroclaw Medical University, T. Chałubińskiego 10, 50-368 Wrocław, Poland; aleksandra.kuzan@umed.wroc.pl (A.K.); kamilla.stach@umed.wroc.pl (K.S.); lukasz.kotyra@umed.wroc.pl (Ł.K.); irena.kustrzeba-wojcicka@umed.wroc.pl (I.K.-W.); 2 Department and Clinic of Internal Medicine, Pneumology and Allergology, Wroclaw Medical University, Marii Skłodowskiej-Curie 66, 50-369 Wrocław, Poland; karolina.nowakowska@student.umed.wroc.pl; 3 Department of General, Minimally Invasive and Endocrine Surgery, Wroclaw Medical University, Borowska 213, 50-556 Wrocław, Poland; krzysztof.kaliszewski@umed.wroc.pl (K.K.); pawel.domoslawski@umed.wroc.pl (P.D.); 4 Department of Immunology of Infectious Diseases, Hirszfeld Institute of Immunology and Experimental Therapy, Polish Academy of Sciences, Weigla 12, 53-114 Wrocław, Poland; andrzej.gamian@hirszfeld.pl

**Keywords:** thyroid gland pathology, glycation, AGEs, RAGE, MAGE, oxidative stress

## Abstract

The patho-mechanism of changes in the thyroid gland, including carcinogenesis, is a complex process, which involves oxidative stress. The goal of our investigation was to verify the extent of stress in the thyroid gland related to glycation. The study samples were comprised of blood sera, thyroid, and adipose tissue sections probed from 37 patients diagnosed with thyroid cancers and goiter. Using immuno-enzymatic and fluorometric assays we analyzed the content of advanced glycation end-products (AGEs), pentosidine, receptors for advanced glycation end-products (RAGE), scavenger receptor class (SR)-A, SR-B, glutathione, malondialdehyde and nitric oxide synthase. In addition to classic AGEs, a recent study detected the melibiose-derived glycation (MAGE) product. We demonstrated the presence of AGEs, MAGE and their receptors of the RAGE and SR-A. In addition, in the control samples of thyroid glands SR-B groups were detected as well as of pathological groups without noticeable tendency to antigen concentration in the area of carcinogenesis. Fluorescent AGEs correlate positively with glutathione, which supports the assumption that glycation stress leads to augmentation of oxidative stress and increase of the intensity of antioxidant mechanisms.

## 1. Introduction

The primary function of the thyroid gland is the production, storage, and release of hormones to the blood. The thyroid parenchyma primarily consists of vesicles lined with a single-celled cubic epithelium, in which three hormones are synthesized: T3 (l-3,5,3′-Triiodothyronine), T4 (Thyroxine, l-3,5,3′,5′-Tetraiodothyronine) and C cells responsible for calcitonin production [1].

Based on Sherman [2], thyroid cancer accounts for about 1% of de novo detected malignant tumors, and women were documented at higher risk to succumb from cancer than men (1.5% vs. 0.5%). Thyroid cancer may occur at any age, but it is rare in childhood. Most tumors are diagnosed during the third to sixth decade of life. Thyroid cancer accounts for 25% of all malignancies diagnosed in young people between 20 and 40 years old [3].

Most thyroid cancers are benign tumors—adenomas. Only 10% of thyroid tumors are malignant. The World Health Organization (WHO) estimates that nodular goitre occurs in about 12% of the population, and it is from 2 to 10 times more common in women. In Poland, the prevalence of goitre is estimated at around 8%, and in other European countries this occurrence is lower [4].

Recently, glycation has been reported to be associated with thyroid pathology. It is found that the thyroid gland does not perform properly in the state of hyperglycaemia [5]. There are single papers documenting the role of glycation factors such as methylglyoxal in thyroid oncogenesis [6]. Up to date, little is known on advanced glycation end products (AGE) in patients’ blood sera. A few scientific papers only present measures of the content of glycated haemoglobin (HbA1c) [5], which in fact is not an advanced glycation end product but only an Amadori product. Glycated haemoglobin is formed during the early steps of the glycation [7]. The content of AGEs from the thyroid gland samples after thyroidectomy is usually unassessed. Further study is therefore necessary in this field, which is one of the aims of this paper.

A new type of AGE has recently been synthesized in vitro in anhydrous conditions, namely melibiose-derived advanced glycation end-product (MAGE). It is reported that this imitates a unique epitope present in human and animal tissues [8]. We perform here an initial work to verify whether this novel glycation adduct may participate in thyroid pathology.

The best known and most widely described class of receptors for AGE products are RAGE receptors, whose role in many pathological conditions of the body is increasingly emphasized. Scavenger receptors (SR) are the second receptor group for AGEs. They represent a large family of proteins that are structurally distinct and contribute to a wide range of biological functions. The next significant type of AGE receptor is lectin galectin-3 (Gal-3), one of the most frequently studied immunomarkers in the field of thyroid pathology [9]. Interaction of AGEs with RAGE receptors generates reactive oxygen species (ROS) and, as a result, leads to oxidative stress [10]. Oxidative stress is a phenomenon in which overproduction of highly reactive oxygen and nitrogen compounds leads to maladies in the structure of proteins and DNA, and to lipid peroxidation. 

The aim of our study is the analysis of the influence of the presence of oxidative stress and glycation products, together with their receptors. on the patho-mechanism of changes in the thyroid gland.

## 2. Materials and Methods

### 2.1. Materials

The surgical thyroidectomy tissue samples and sera from patients treated in the First Department and Clinic of General, Gastroenterological and Endocrine Surgery at Wroclaw Medical University were subjects in our study. Patients were enrolled between May 2017 and June 2018. Those who were eligible for the study signed informed consent to participate. The Bioethical Commission at the Wroclaw Medical University approved the study (opinion number KB-430/2017). All the measures were performed in agreement with the ethical standards of the Helsinki Declaration. Informed consent was obtained for research on human samples.

The analysed group consisted of 37 patients, including 6 men and 31 women from the age of 25 to 75 (average = 55.5 years; SD = 13.7 years). Tumors occurring in 29 patients were diagnosed with goitre, and out of four papillary cancer cases, one was squamous cell cancer and three were adenoma carcinoma. In all cases, the diagnosis was verified by patho-morphological screening. 

Four types of sample were taken from each patient:

(a) blood for serum extraction.
(b) tumor sample.
(c) section of tissue surrounding the tumor considered to be a control sample.
(d) sample of adipose tissue surrounding the thyroid gland.

Paraffin slides of tissue samples were prepared. The serum was frozen and stored at −80 °C temperature. 

The patients’ blood was tested for the following parameters: thyroid stimulating hormone (TSH), free thyroxine (FT4), haemoglobin (HGB), creatinine. According to their medical archives none of the 37 examined patients suffered from diabetes. There were also no cases of renal diseases in the examined group—the creatinine level stayed within the normal range (average = 0.72 mg/dL, SD = 0.11 mg/dL). Characteristics of the study group is presented in Table 1. 

### 2.2. Immunohistochemistry

The slides were deparaffinised, rehydrated and rinsed twice in phosphate-buffered saline for 10 min. The antigen retrieval with HistoReveal (Abcam, Cambridge, England) was performed as a pre-treatment. Slides were incubated with 3% hydrogen peroxide for 10 min and then with Protein Block (Abcam) for 30 min. Sections were incubated with IgG anti-AGEs mouse monoclonal antibodies (1: 300, MyBioSource, San Diego, California, USA), IgG anti-RAGE rabbit polyclonal antibodies (1:300; Abcam), IgG anti-SRBI rabbit monoclonal antibodies (1:100, Abcam), IgG anti-CD204 (SR-A) rabbit polyclonal antibodies (1:150, Abcam) and IgG anti-NOS-3 mouse monoclonal antibodies (1:50, Santa Cruz, Santa Clara, USA) at 4 °C for 18 h. The colour reaction was performed using Biotinylated Goat Anti-Mouse Antibodies, Streptavidin Peroxidase and DAB substrate (ab64264, Abcam). Nucleic and other basophilic proteins were counterstained with haematoxylin. The negative control consisted of samples incubated without the primary antibody. Slides were dehydrated, closed with a glass coverslip using DPX (Aqua Medica, Lodz, Poland) and examined using an Olympus BX51 microscope. The content of antigens was assessed with the half quantitative method, assessing the intensity and extent of immunohistochemical reaction in a four-degree scale: [–] no visible product in the sample, [+] slight intensity and extent of reaction, [++] intermediate intensity—about 50% of slide contains colorimetric product of immuno-enzymatic reaction, [+++] immuno-enzymatic reaction is complete in most cells and it is very intense (lots of dark reaction product). 

### 2.3. ELISA—Competitive Test

96-well MaxiSorp (Nunc®, Sigma-Aldrich, Darmstadt, Germany) plates were covered with synthetic MAGE based on myoglobin, obtained by high temperature microwave synthesis performed in anhydrous conditions. The coating process lasted 4 h at 37 °C temperature. In the next step the plates were blocked with 10% skim milk for 18 h at 4 °C. 50 μL serum sample was taken to the Eppendorf test tubes, diluted twice with PBS and finally 150 μL of non-commercial monoclonal IgE antibodies anti-MAGE were added. Equivalent to the serum samples, a standard of low molecular mass MAGE (LMW-MAGE, noncomercial, obtained as described in [8]) prepared also by high temperature microwave synthesis, was added in the range of 0–30 µg/100 µL to, analogously to the serum monoclonal antibodies. Antibodies and standards are described in [8]. The incubation with antibodies was conducted in tubes for 45 min, then samples were transferred to coated plate and incubated for 1.5 h. The plate was rinsed three times with PBST and subsequently a solution of secondary antibody—(polyclonal to mouse IgE (Fc specific)-peroxidase (HRP) (1:6000, OriGene,**** Rockville, MD, USA) was added and incubated for 1.5 h at room temperature. Then peroxidase was added to react with the substrate—*o*-phenylenediamine hydrochloride (OPD, Sigma-Aldrich, Darmstadt, Germany) and absorbance at 450 nm was read with Enspire plate-reader (Perkin Elmer, Waltham, MA, USA). Based on the standard curve we calculated the content of glycation products in each sample.

### 2.4. The Analysis of Fluorescent AGEs and Pentosidine Content Using the Fluorometric Method 

The content of fluorescent AGEs was measured using their fluorescent properties. The methodology was a slight modification of that of Leszek et al. [11]. Serum samples were diluted 100-fold with 0.9% NaCl. The absorbance of these samples at λ = 280 nm were measured using quartz plate (Hellma Analytics, Müllheim, Germany) on EnSpire Multimode Plate Reader (Perkin Elmer). The fluorescence of samples at excitation wavelength 370 nm and emission 440 nm were measured using black plates (Thermo Fisher Scientific, Waltham, MA, USA) on EnSpire Multimode Plate Reader to assess the content of total fluorescent AGEs. The number of flashes was 100. To assess the total pentosidine content in the sample, the fluorescence of the samples was measured analogously at excitation wavelength 335 nm and emission 385 nm. Fluorescence was divided by Abs 280 assuming the resulting values as data expressed in arbitrary units.

### 2.5. The Assessment of Content of Compounds with Thiol Groups (Glutathione)

Determination of glutathione (one of main factors responsible for the antioxidative mechanism in the body) concentration is usually performed as a measure of thiol groups content using Ellman’s reagent (DTNB). The method was developed in 1991 by Rice-Evans et al. [12]. Reaction is performed between compounds containing thiol groups from patients’ sera and 0.1 M DTNB in 10 mM sodium-phosphate buffer pH 8.00, in the presence of 10% sodium dodecyl sulfate (SDS). Parallel to this we performed the reaction with glutathione of concentration 0.25–1 mM to draw a standard curve. The reaction was conducted at 37 °C for 60 min. In the next step we measured absorbance against blank sample at λ = 412 nm and based on the standard curve, we calculated the content of thiol groups in the analysed material.

Assessment of lipid peroxidation based on determination of malondialdehyde (MDA) in serum is done with the method of reaction of MDA with thio-barbituric acid (TBA). The pink product was later determined by spectrophotometry [13]. Reactive mixtures consisted of serum, 15% TCA acid solution in 0.25 M HCl and 0.37% TBA in 0.25 M HCl. The mixtures were incubated for 20 min at 100 °C then cooled down, centrifuged for 5 min at 4700 RPM. Absorbance of the supernatant was measured at 535 nm. Calculation of MDA concentration was performed using Lambert-Beer law, at absorbance coefficient ε = 156 mmol^−1^ L cm^−1^. 

### 2.6. Statistical Analysis

A statistical evaluation and graphic representation of the obtained results were carried out using the GraphPad Prism 8 (GraphPad Software, Inc., San Diego, CA, USA), and Statistica 13.3 (TIBCO Software Inc., Palo Alto, CA, USA) software. For each pair of performed tests, the correlation coefficient r-Pearson and statistical significance were calculated. For the comparison of the groups, the Kruskal–Wallis test by ranks was used. Statistic relevancy was at *p* < 0.05. 

## 3. Results

### 3.1. Localization and Semiquantitative Analysis of AGEs, Receptors for Advanced Glycation End-Products (RAGE, SR-A, SR-B) and Nitric Oxide Synthase (NOS-3) in Samples of Thyroid Gland and of Adipose Tissue 

**Figure 1:**
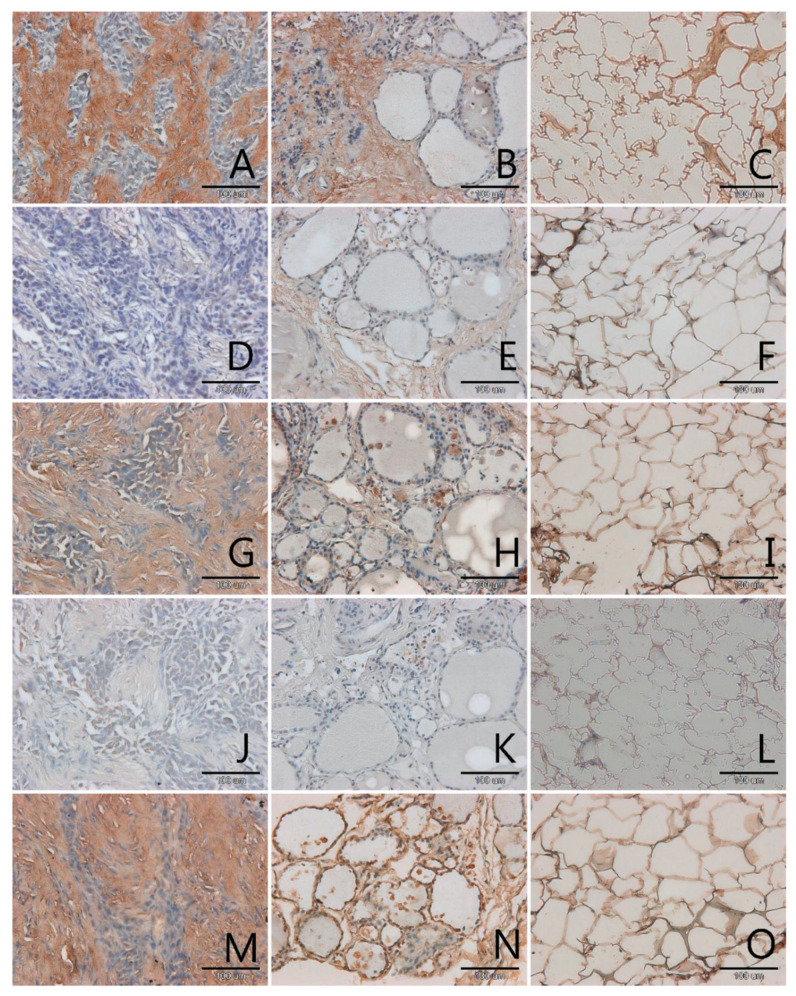
Immunohistochemical detection of advanced glycation end-products (AGEs) (**A**–**C**), receptors for advanced glycation end-products (RAGE) (**D**–**F**), scavenger receptor class (SR)-A (**G**–**I**), (SR)-BI (**J**–**L**) and nitric oxide synthase-3 (NOS-3) (**M**–**O**) in sections of the thyroid gland with papillary cancer (**A**,**D**,**G**,**J**,**M**), in the paired control sections (**B**,**E**,**H**,**K**,**N**) and in the fatty tissue surrounding the tumor (**C**,**F**,**I**,**L**,**O**). Magnification 200× (scale bar 100 µm).

**Figure 2:**
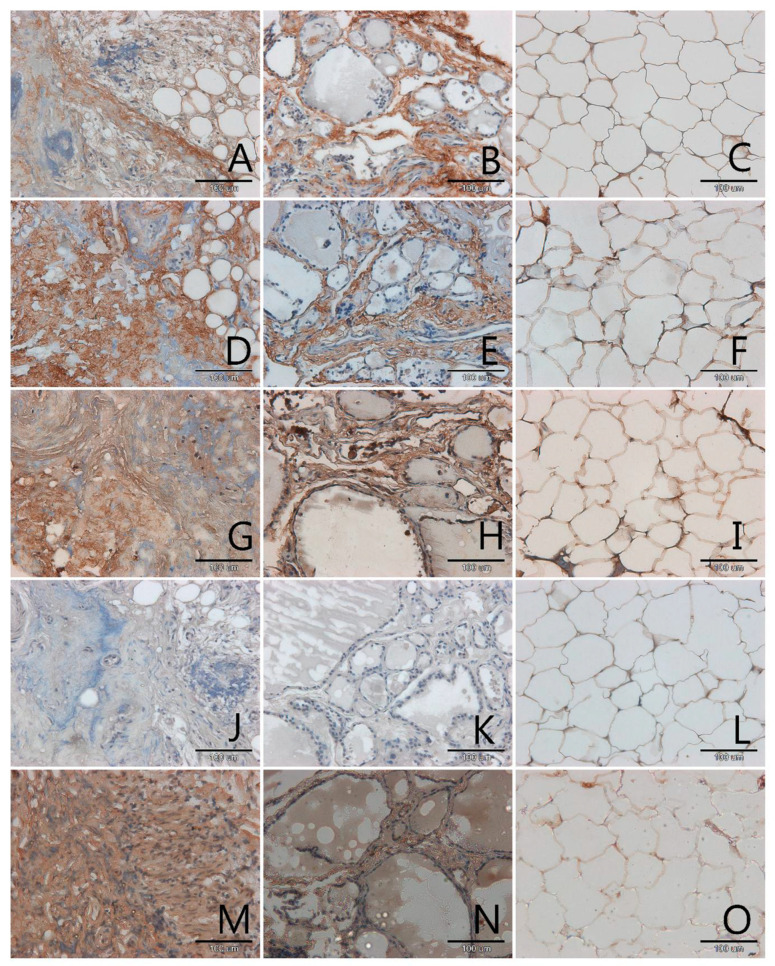
Immunohistochemical detection of AGEs (**A**–**C**), RAGE (**D**–**F**), SR-A (**G**–**I**), SR-BI (**J**–**L**) and NOS-3 (**M**–**O**) in sections of the thyroid gland with squamous cell carcinoma (**A**,**D**,**G**,**J**,**M**), in the paired control sections (**B**,**E**,**H**,**K**,**N**) and in the fatty tissue surrounding the tumor (**C**,**F**,**I**,**L**,**O**). Magnification 200× (scale bar 100 µm).

**Figure 3:**
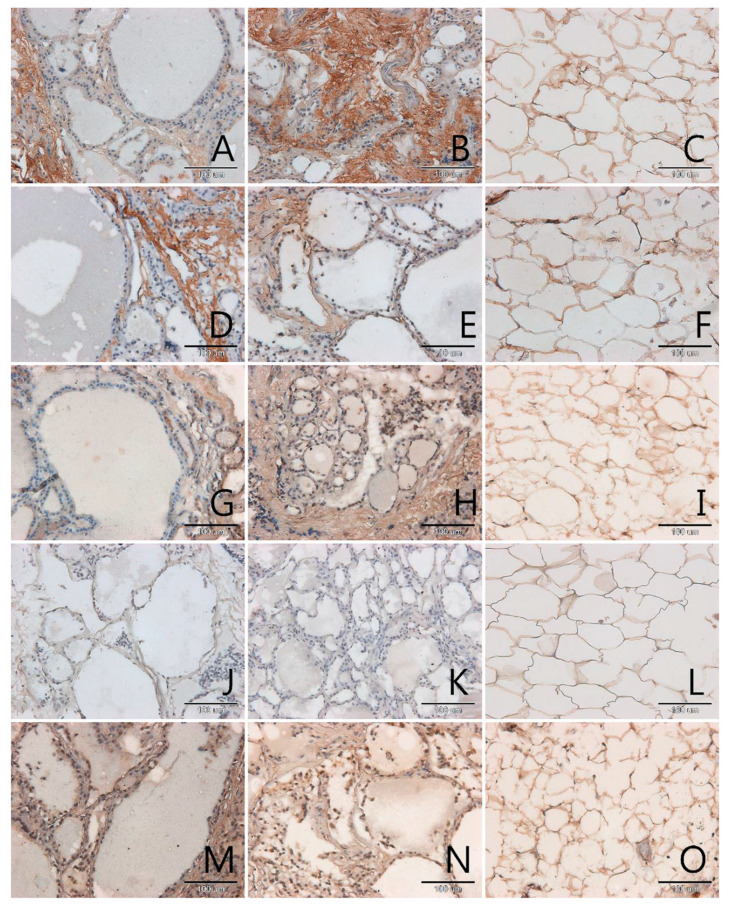
Immunohistochemical detection of AGEs (**A**–**C**), RAGE (**D**–**F**), SR-A (**G**–**I**), SR-BI (**J**–**L**) and NOS-3 (**M**–**O**) in sections of the thyroid gland with nodular goitre (**A**,**D**,**G**,**J**,**M**), in the paired control sections (**B**,**E**,**H**,**K**,**N**) and in the fatty tissue surrounding the tumor (**C**,**F**,**I**,**L**,**O**). Magnification 200× (scale bar 100 µm).

**Figure 4:**
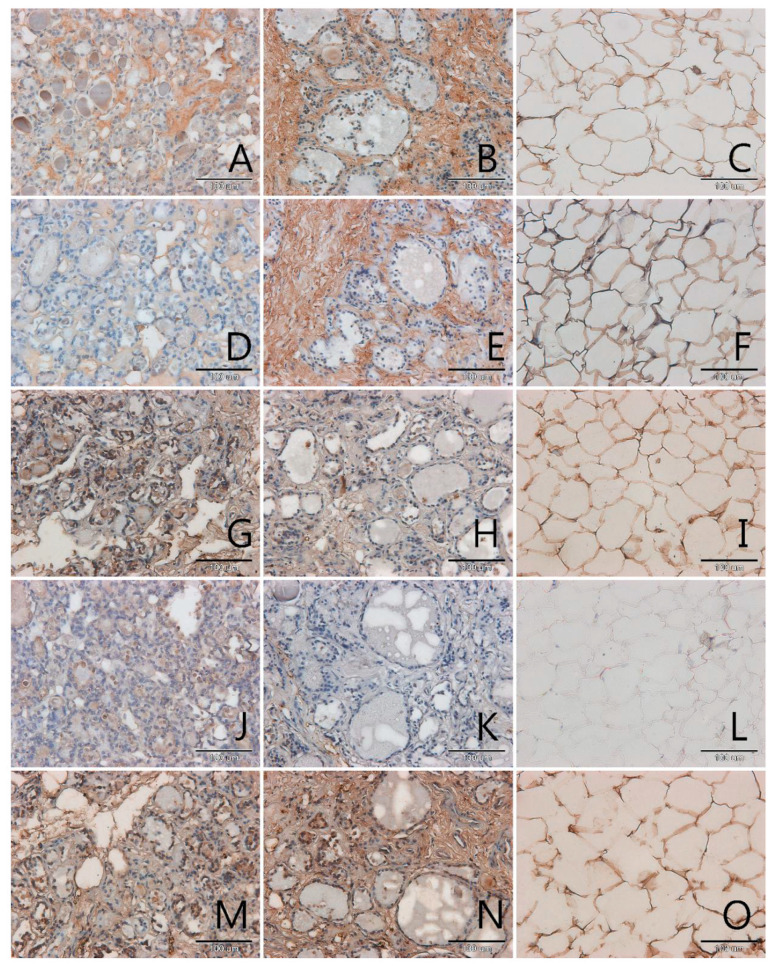
Immunohistochemical detection of AGEs (**A**–**C**), RAGE (**D**–**F**), SR-A (**G**–**I**), SR-BI (**J**–**L**) and NOS-3 (**M**–**O**) in sections of the thyroid gland with follicular adenomas (**A**,**D**,**G**,**J**,**M**), in the paired control sections (**B**,**E**,**H**,**K**,**N**) and in the fatty tissue surrounding the tumor (**C**,**F**,**I**,**L**,**O**). Magnification 200× (scale bar 100 µm).

AGEs occurred mainly in the extracellular matrix in all samples, pathological and control (Figure 1, Figure 2, Figure 3 and Figure 4, panels A–C). Only in a few samples were AGE antigens present in follicular cells (Figure 4, panel B). Comparing samples of healthy thyroid tissue and samples of tumors, we concluded that there are slightly more AGEs in control groups than in tumors. We suspect that this is because of the larger presence of extracellular matrix proteins in control groups than in pathological tissue. Comparing different kinds of thyroid pathology, no differences between groups were noticed. 

RAGE receptor co-localizes clearly with AGE. In some cases, this antigen is characterised by some kind of diversity in occurrence, depending on tissue type: pathological or control. In two out of three analysed cases of adenoma the content of RAGE is significantly higher in control samples than in tumor samples (Figure 4, panel D,E). In tumor goitre such dependence is observed in seven cases. In the sixth, it is quite the opposite—segments of tissue from outside the tumor seem to have fewer RAGE than the segment of tumor itself (Figure 3, panel D,E). In the remaining 16 cases classified as tumor goitre we did not observe any difference in degree of intensity of immunohistochemical reaction between sample of healthy thyroid gland and tumor. This was similar in the case of papillary (Figure 1, panels D,E) and squamous cell carcinoma (Figure 2, panel D,E). On the basis of analysis, we concluded that there was no distinct pattern in RAGE occurrence in analysed tissues, which disqualifies this antigen from consideration as a potential marker for oncological thyroid diseases.

Scavenger receptor class A (SR-A) occurs in endothelium and in interfollicular macrophages (Figure 1, Figure 2, Figure 3 and Figure 4, panels G,H). In samples with diagnosis of papillary cancer it also occurs in cancer cells and around the extracellular matrix (Figure 1, panel G). We also observed a significant expanse and immunohistochemical reaction intensity in case of samples with squamous cell carcinoma (Figure 2, panel G). 

Scavenger receptor class B (SR-B) is expressed to a relatively small degree in the thyroid gland. In some paraffin slides with thyroid segments a lack of antigen was observed—these were mainly in segmenting of tumor goitre (Figure 3, panel J). There was a significantly increased expression of SR-B noticed in segments of papillary carcinoma (Figure 1, panel J). In the papillary type of cancer, SR-B occurs in the cytoplasm of mutated follicular cells and captures the entire surface of cytoplasm apart from the area occupied by the characteristic nucleus, although the intensity of immunohistochemical reaction is not high (Figure 1, panel J). This is similar with adenomas (Figure 4, panel J). We highlighted that SR-B antigen occurrence in samples deriving from the same patient was coherent—if the SR-B antigen was present in a segment of thyroid tumor, it occurred in the control sample as well (Figure 1, panels J,K, Figure 4, panels J,K).

In our study we also proved that endothelial nitric oxide synthase was expressed in the thyroid gland. High NOS-3 immunoreactivity was found in the cytoplasm of some follicular cells as well as in endothelial cells in thyroid (Figure 1, Figure 2, Figure 3 and Figure 4, panels M,N). We did not observe significant differences between kinds of thyroid pathology and differences between control samples and tumor segments.

In adipose tissue we observed the presence of all analysed antigens: AGEs, RAGE, SR-A, SR-B and NOS-3, although in some cases the intensity of immunohistochemical reaction was very low, especially in case of SR-B. The localization of the tested antigens was mainly in membrane. In few nuclei of adipose tissue, no examined protein was observed. The glycation products, as with many other tissues, were intensively stained in areas of extracellular matrix accumulations (Figure 1, panel C). We expected an observation of high amount of scavenger receptors on the surface of adipocytes membrane and we observed this result for SR-A (Figure 1, Figure 2, Figure 3 and Figure 4, panels I,L).

### 3.2. The Analysis of Correlation between Distinct Biochemical Parameters 

**Figure 5:**
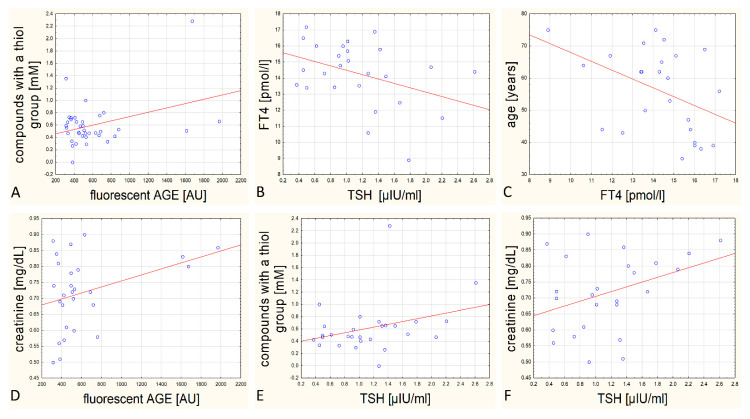
Graphs of dispersion of results for pairs of analysed parameters in blood serum. Panel (**A**)—dependency between the content of compounds with thiol groups and fluorescent AGEs, panel (**B**)—dependency between concentration of FT4 and TSH, panel (**C**)—dependency between patient’s age and FT4 concentration, panel (**D**)—dependency between creatinine level and amount of fluorescent AGE products in serum, panel (**E**)—dependency between compounds with thiol group and TSH, panel (**F**)—dependency between concentration of creatinine and TSH. For data from panels (**A**–**C**) we proved statistically relevant correlation.

The comparison of biochemical parameters of blood of patients taking part in the examination have led to statistically significant correlations between:

1. Content of fluorescent AGEs and thiol groups—statistically, the more AGEs in the patient’s blood, the higher the concentration of compounds with thiol group in their blood serum, including glutathione (r = 0.361, *p* = 0.028; Figure 5, panel A);
2. Concentration of TSH and FT4—statistically, the higher the concentration of TSH, the lower level of FT4 hormone in the blood serum (r = −0.395, *p* = 0.046; Figure 5, panel B);
3. Concentration of FT4 and age—statistically, the older the patient, the lower the level of FT4 hormone in the blood serum (r = −0.427, *p* = 0.03; Figure 5, panel C).

Additionally, several different pairs of parameters are close to obtaining statistical relevance:

1. Correlation between creatinine concentration and the content of fluorescent AGEs in blood serum, (r = 0.352, *p* = 0.071; Figure 5, panel D);
2. Correlation between the content of compounds with thiol groups and TSH in blood serum (r = 0.335, *p* = 0.082; Figure 5, panel E);
3. Correlation between creatinine concentration and TSH concentration in blood serum (r = 0.369, *p* = 0.070; Figure 5, panel F).

We assume that the results would indicate statistical relevance if the examined group of patients were bigger. Statistics is also disturbed by single distinctive results. In case of correlation between thiol group compounds content and TSH we obtained one distinctive result (Figure 5, panel E). Similarly, in case of correlation between creatinine concentration and content of fluorescent products of advanced glycation we obtained in statistical analysis three distinctive results (Figure 5, panel D). 

**Figure 6:**
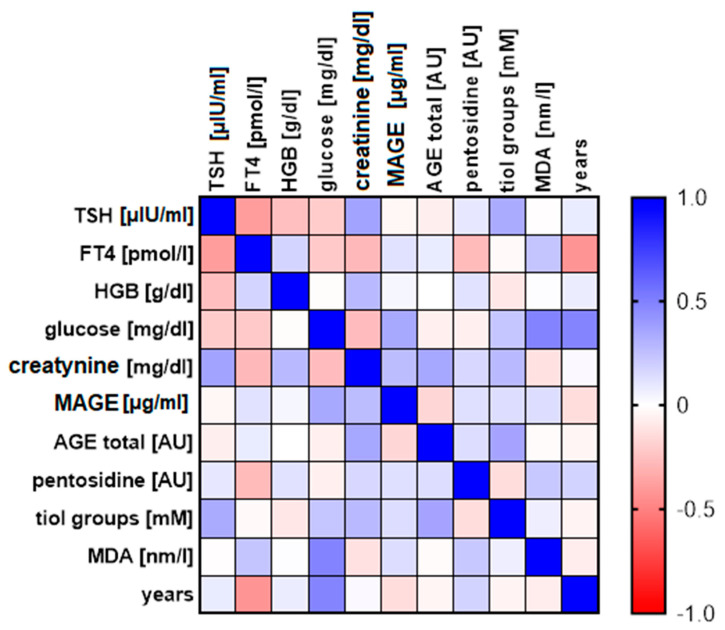
The matrix of correlation for marked biochemical parameters and patients’ age. The scale in range from −1 to 1 shows Pearson coefficient.

Below we present a picture comparing all the parameters with the scale of correlation power (Figure 6) and the table representing all relations between analysed parameters (Table 2). 

**Figure 7:**
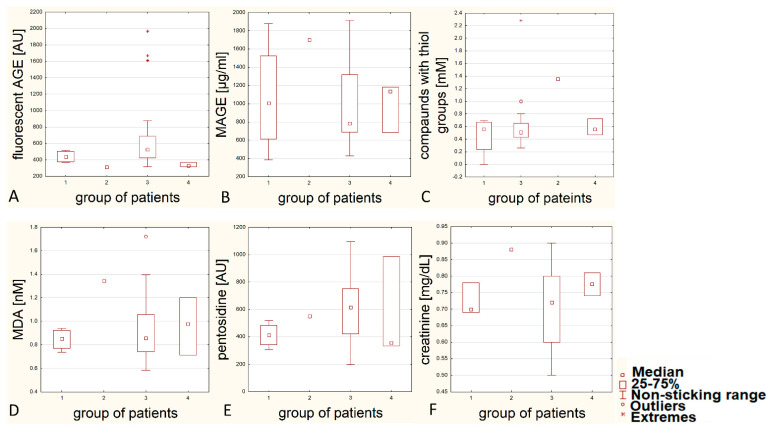
Comparison between patients’ groups and levels of fluorescent glycation products (panel **A**), MAGE (panel **B**), compounds with thiol group (panel **C**), malondialdehyde (panel **D**), pentosidine (panel **E**) and creatinine (panel **F**) in blood serum. Groups: 1—patients with papillary carcinoma, 2—patients with squamous cell carcinoma, 3—patients with goitre, 4—patients with adenoma.

We also analysed the differences between examined parameters in relations to thyroid pathology type using boxplots (Figure 7). Unfortunately, the differences between the groups, probably due to the insufficient sample size, are statistically insignificant, but the graphic representation shows some tendencies (Figure 7). The average content of fluorescent AGEs, MAGE, compounds with thiol group, MDA, pentosidine and creatinine in patients’ serum with papillary carcinoma does not differ from results of patients with benign tumors, which means tumor goitre and adenomas. In the serum of patient with squamous cell carcinoma we found more AGE10, less fluorescent AGEs and more compounds with thiol group that in the other patients. The patient with squamous cell carcinoma has also got an outstandingly high creatinine concentration (0.88 mg/dL) and quite high MDA level in serum (1.3 nm/L). 

The data considering just one case (squamous cell carcinoma) can be treated only tentatively and the conclusions cannot be generalised to any scope.

In our analysis, three patients with diagnosed tumor goitre are noteworthy because of the extremely high number of fluorescent AGEs in blood serum compared to other patients (so called extrema in Figure 7, panel A). Interestingly, one of those patients (number 14) is characterised by high level of compounds with thiol groups (Figure 7, panel C). We can assume in this patient an extraordinary metabolic disorder, connected with intensive oxidative stress, diabetes and/or atherosclerosis. Unfortunately, there is no more data which makes it possible to conduct a further analysis and interpretation of our suspicion.

## 4. Discussion

### 4.1. Participation of Glycation in Thyroid Pathology

The presence of AGEs, RAGE and scavenger receptors in the thyroid gland is rarely mentioned in the literature. To our recent knowledge, we are the first to compare three different receptors for AGEs: RAGE, SR-A, SR-B in four types of thyroid pathology: papillary carcinoma, squamous cell carcinoma, goitre and adenoma. 

The glycation reaction which results in AGE formation is strongly associated with diabetes mellitus (DM). In the state of hyperglycaemia, a high concentration of sugars, i.e., glycation substrates, intensifies the process. DM and thyroid diseases are the two most common endocrine disorders encountered in clinical settings. Furthermore, there is a deep underlying relation between DM and thyroid dysfunction. Thyroid diseases are often caused by oxidative stress and are related to occurrence of too high concentrations of AGEs in the body [14]. In our assessed group of patients, no one was diagnosed with diabetes. In our view it is worthy to carry out further studies on AGEs and their receptors’ participation in pathological changes of thyroid gland on a bigger group of patients including patients with diabetes. 

Pentosidine is one of the best-known AGEs. It is formed as a consequence of the reaction of a sugar with a lysine residue and the formation of a reactive adduct that, reacting with arginine, cross-links the proteins that contains the two amino acids. Pentosidine correlates with the presence and severity of diabetic complications [15]. The plasma concentration of pentosidine is shown as biomarker, for example, for age-related macular degeneration [16], progression of diabetic nephropathy [17] or as excellent specific biomarker for bone quality. Tomizawa [18], Kawaguchi [15] et al. presented pentosidine as a biomarker of ossification of the spinal ligament. So far the reports on relationship between pentosidine in blood serum content and pathological changes in the thyroid gland have not yet been published. Our study indicates that pentosidine content in blood is not associated with any of the analysed thyroid pathologies. 

Results of quantitative analysis obtained by our study indicate that AGEs occur mainly within extracellular matrix. This is not astonishing, as extracellular matrix proteins like collagen are particularly susceptible to AGE modification because of slow turnover rates [19].

In our study, we investigated AGEs with two methods—fluorometric and immuno-enzymatic. The obtained results do not correlate with one another but require debate. First and foremost, attention should be paid to the fact that quantitative determination of AGEs in biological material is challenging in terms of approach. The problem is caused by the variety of compounds for analysis. The number of AGEs in biological material directly relates to conditions regarding how the products are formed (pH, the number of free radicals, metal ions and so on) and on the type and concentration of substrates of the reaction. Moreover, AGEs occur naturally in living organisms in small amounts. The isolation of AGEs from tissues may lead to undesirable chemical modifications. The easiest and most popular method of estimation of AGEs in biological material is based on specific spectrophotometric and fluorescent properties of these compounds. It must be highlighted, though, that a part of measured fluorescence can come from desmosine and iso-desmosine, which fluoresces at the same wavelength as the one in the analysis [20]. The shown situation deals mainly with tissues, not serum, because desmosine and its derivatives are ingredients of elastin which is almost absent in blood. The spectrophotometric method is not able to specify the structure of analysed AGEs in the sample. Immunochemical techniques, which use antibodies against specific epitope, are more precise for determination of AGEs. Some scientists immunize animals with more complex epitopes. Ling et al. [21] developed two original antibodies: the first is anti-“fluorescent linker”, whose epitope consists of two lysine residues cross-linked by pyridine fluorophore which consists of two glucose residues. The second antibody patented by these authors was produced after immunization of mouse with AGE-RNAse conjugate [21]. In their research they chose positive clones for AGE-BSA, but negative for CML-BSA and RNase. We present here only a fragment of the possibilities which immunochemistry can offer in case of determination of antigens typical for AGEs. AGEs are characterised by a remarkable variety and complexity of chemical properties. 

In this paper we chose to use non-commercial monoclonal anti-MAGE antibodies, obtained according to the method described by Gamian et al. [8]. Anti-MAGE antibodies are unique, because they do not react with proteins modified by different α-oksoaldehydes, or monosaccharides. The analysis of obtained data does not allow us to claim that there are any correlations between the content of antigen MAGE and different studied biochemical parameters, or even a relationship between the amount of this antigen and the amount of fluorescent AGEs. We assume that MAGE corresponds to an entirely different pool of AGEs, different from the mixture of fluorescent AGEs. The comparison of the content of MAGE with thyroid pathology type allows the conclusion that serum level of this antigen seems to be higher in the patients with squamous cell carcinoma than in patients with benign adenomas or tumor goitre. We also noticed that there is more MAGE in segments of thyroid gland with squamous cell carcinoma in comparison with papillary carcinoma, but this is not statistically verified. Further studies are being conducted in order to gain understanding of MAGE content in different human tissues in relations to disease [19,22].

### 4.2. RAGE Receptors in Thyroid Pathology

It is suggested that RAGE is also the major receptor for AGEs in the thyroid gland. Its presence is potentially significantly linked with inflammation. Caspar–Bell et al. [10] obtained remarkable results, which are the basis for the conclusion that low levels of sRAGE and high levels of AGEs would generate more ROS leading to hyperthyroidism and its complications [10]. Prasad [23] presents an opinion that AGEs/sRAGE may be a universal risk marker for different diseases. In his analysis the serum levels of AGEs and ratio of AGEs/sRAGE were higher in all types of analysed patients (patients with hyperthyroidism, non-ST-elevation myocardial infarction, thoracic aortic aneurysm and hypercholesterolemia) compared to healthy control subjects [23]. However, we do not have measured sRAGE, but only of membrane receptors in tissue, AGEs in tissue and serum. Nevertheless, our results do not confirm that AGEs or RAGE were present in a greater amount in samples of pathologically altered thyroid than in control samples, independent of the type of tumor.

### 4.3. Scavenger Receptors in Thyroid Pathology

The next receptors to be analysed for AGEs were scavenger receptors type A and B. There is far less SRB-I antigen in the studied material than SR-A receptors and RAGE. One should consider that immunohistochemistry is a semiquantitative method and acquired results can be connected with different degrees of affinity between antibody and antigen. Scavenger receptors have a large variety of ligands and for this reason it is difficult to assess whether their presence is connected with glycation, lipid metabolism, apoptosis or another phenomenon affecting the appearance of their ligands.

The link between the thyroid and SRB-I is that FT3 may increase the content of scavenger receptor B-I in the thyroid gland and promote the transport of cholesterol to the liver. The potential role of scavenger receptors in the expansion of cancer is also established, despite the fact that nobody had analysed thyroid cancer in this context. It is found that overexpression of SRB-I occurs in human choriocarcinoma cells, malignant human epithelial cells, prostate cancer cells, hepatoma cells and breast cancer cells. In the case of the latter, SRB-I protein levels were found to be significantly elevated in malignant tissues compared with surrounding histologically disease-free tissues [24]. We did not observe such a phenomenon in the cancers analysed by our team. Yu et al. [25] proved that SRB-I can partially be pro-oncogenic and partially anti-oncogenic, at least in the case of breast cancer. Overexpression of SRB-I protects cells against TNF-α-induced apoptosis, whereas expression only of the extracellular domain of SRB-I significantly inhibited the cell proliferation [25]. SR-A has been assigned a pro-cancer role, mainly by mediating excessive lipid accumulation, which may deregulate dendritic cells’ differentiation toward a tolerogenic phenotype in the tumor microenvironment. Disturbed differentiation of dendritic cells suppresses the functions of effector T cells and promotes the evasion of cancer from immune attack. SR-A also inhibits the antigen-cross-presenting functions and activation of dendritic cells. Furthermore, SR-A contributes to tumor progression, invasion and metastasis via interacting with Mer tyrosine kinase and as a result of Tumor-Associated Macrophages’ (TAMs) polarization toward an M2-like phenotype [25].

In our study we did not observe SR-A to be significantly more abundant in oncogenic samples than in control. It is also impossible to differentiate cancer types by analysis of SR-A content in tissue samples.

Particularly in view of scavenging receptors, we also analysed adipose tissue surrounding the pathologically altered thyroid. In accordance with our anticipations, we found a particularly great amount of SR-A, which is mainly responsible for LDL intake, as well as its modified forms. On the other hand, there were only few SRB-I, which binds HDL—“the good cholesterol”. This may prove that there is an unsuccessful lipoprotein turnover in the thyroid gland.

### 4.4. Discussion of Results Considering Thyroid Hormones, Creatinine and Patients’ Age

The mechanism of thyroid hormone regulation based on negative feedback loop is well known and it is common knowledge that thyroxine is mainly responsible for reversible inhibition of TSH excretion. In the conducted study we obtained negative correlation between TSH and FT4. Our results are consistent with the results of Du et al. [26]. 

It is also quite well known that physiological aging is associated with mild deficiency of thyroid function or subclinical hypothyroidism. As expected, we observed a negative correlation between FT4 and age. Other researchers, such as Park [27] and Del Buono [28] obtained a similar relation between thyroid hormones. Del Buono claims that FT3/FT4 ratio could be predictive of a predisposition for the expansion of cancer, chronic diseases and the degree of disability [28]. Creatinine level in blood serum is used for evaluation of renal function. This parameter is routinely determined before thyroidectomy. The patients we evaluated had creatinine levels within the norm. We found positive correlations between creatinine, TSH and fluorescent AGEs, close to statistical relevance. It is known that build-up of AGEs damages kidneys and leads to nephropathy [24]. AGEs concentration in serum seems to be a very sensitive indicator of changes in kidneys. Creatinine seems to be associated with AGE content even within the norm. Assumptions on this topic should be postponed until more research is done on a larger number of samples, particularly on samples from patients with confirmed nephropathy. It is thought that TSH might be elevated in nephrotic patients compared to control healthy people, which in a comparable way can be interpreted as in the previous case. The potential tendency that we noticed in our research is consistent with other researchers’ results [29].

Thyroid hormones synthesis is physiologically connected with formation of ROS. The more TSH, the more glutathione is needed, which could stimulate thiol groups in oxidised proteins. Based on our results TSH- glutathione relation is a positive correlation of average power, close to obtaining statistical relevancy, which is exactly what we anticipated. We could not find any scientific paper that shows a similar correlation on group of patients with pathological thyroid gland. The correlations presented here are unique, but there is essential to continue the research on more numerous groups of patients.

### 4.5. Oxidative Stress in Pathomechanism of Thyroid Diseases

In 1990 scientists reported on the occurrence of ROS in oncogenic tissue. In the material coming from oncogenic changes an increase of MDA level was observed as well as decrease of enzymatic antioxidant activity compared to healthy tissues [30]. Below, there is a brief overview of the literature on the subject regarding the relationship of antioxidants and oxidative stress markers in thyroid gland pathology.

Akinci et al. [30] observed a high concentration of Glutathione Peroxidase (GSHPx) and MDA in patients with thyroid carcinoma and after thyroidectomy. Erdamar et al. [31] suggest that in thyroid cancer there is an escalation of lipid peroxidation in tissues and simultaneously a decrease of antioxidant enzymes activity, which makes tissues more prone to the toxic influence of some free radicals [31]. However, there was no significant relationship between different oxidative stress markers and different types of thyroid cancer. Likewise, Du et al. [26] observed that the total level of antioxidants in tissues of papillary cancer and benign goitre did not differ considerably.

Senthil and Manoharan [32] in their experiment observed an increase of lipid peroxidation level and decrease of non-enzymatic and enzymatic antioxidants in the plasma and erythrocytes of 20 patients with papillary thyroid cancer in the second stage. The impairment of defensive mechanisms of antioxidants was claimed to be accountable for intensified lipid peroxidation observed in the plasma and erythrocytes of patients with PTC. The decrease of GSH level, which is a cofactor of glutathione peroxidase but at the same time a powerful antioxidant protecting erythrocytes against damage, was noted in many thyroid cancers. The decrease of activity of glutathione peroxidase can stem from lowered level of reduced glutathione in erythrocytes and plasma. According to Rovcanin [33] and Yu et al. [25], oxidative stress, apart from its physiological role, accompanies thyroid cancers and influences the progression of the disease.

Erdamar et al. [31] demonstrated an increased pro-oxidative activity (lipid peroxidation and impaired defending mechanisms of antioxidants) in tissues taken from patients with goitre and papillary carcinoma. The number of antioxidants was significantly higher in tumor tissue compared to neighbouring healthy tissue and goitre tissue. The presented condition can be found to be a slow process of carcinogenesis, which is often powered by increased oxidative stress. It seems that cancer tissues are more capable of getting rid of free radicals than healthy and benign goitre tissues. Rovcanin [33] suggests that PTC tissue has efficient antioxidative potential to counteract harmful action of free radicals synthesised by the thyroid gland. An increased capability of antioxidative defence can cover up an increased production of free radicals in cancer tissue, which may account for lack of significant differences in overall oxidative cell state between tumor and control tissues. Well-developed vascularity of the thyroid gland allows the getting rid of free radicals and neutralising them in circulation. This is probably the reason that previous research showed an increase of the concentration of oxidizers in serum [34]. 

Donckier et al. [35] showed that the percentage of NOS-3 positive cells in papillary carcinoma cells is greater than in normal thyroid cells. Patel et al. [36] report that average eNOS staining of benign adenomas, multinodular goitres, PTC, FTC and autoimmune lesions was also greater than that of surrounding normal thyroid. The highest expression of eNOS is reported in aggressive cancers [36]. We cannot validate such conclusions. In our samples, the expression of NOS-3 seems to be as high in the case of tissues surrounding the tumor as in the very centre of the lesion. We conclude that endothelial nitric oxide synthase might take part in the development of inflammation of the thyroid gland, but it does not allow differentiation of cancer types. We plan to analyse the expression of inducible NOS as a direction of future research, which may show differences between samples coming from various cancers. 

We proved that fluorescent AGEs and compounds with thiol group, including glutathione, show a positive correlation among one another. Our findings are the first to present a similar effect. We conclude that intensification of glycation results in increase of oxidative stress and antioxidant mechanisms, including synthesis of glutathione. The content of glutathione is exceptionally high in one patient with malignant squamous cell carcinoma, which indicates an increased antioxidative protection in metabolically active malignant tumor.

## 5. Summary and Conclusions

Thyroid pathology is a multifactorial disease. Despite environmental factors, other reasons for the condition may be a genetic predisposition and different metabolic factors, which are connected to the metabolism of sugars and lipids. This study, performed on a group of 37 patients with four different thyroid pathologies, is just an overview of attempts to solve one of the most serious problems in the field of endocrinology.

Our work contains new data on the content of AGEs, pentosidine, RAGE, SR-A, SR-B, glutathione, malondialdehyde and nitric oxide synthase in material from patients diagnosed with thyroid cancers and goitre. According to the current state of knowledge, only a few original papers show the involvement of AGEs and RAGE in thyroid gland pathology.

We described an assessed group that was randomly enrolled. The major restriction of this study was that the groups were unreliable. We acquired five segments of thyroid gland with malignant lesions (papillary carcinoma and squamous cell carcinoma), in ratio with 32 benign lesions (tumor goitre and adenomas). The fact that the groups were not the same in number derives from the natural, far rarer frequency of incidence rate of malignant tumors compared to benign carcinomas. In this connection, results obtained by our study were accumulated in the form of description of some cases of thyroid pathologies. We could not, based on these, write a comparison paper, which would depict the differences between particular types of thyroid change. In this situation our aim was to observe some tendencies, not a characteristic of particular types of benign and malignant cancers. 

The second limitation was that, in the surgery department, patients were not diagnosed for metabolic diseases that could also affect the content of the analytes. Another limitation was the lack of determination of galectin-3. Even the immunohistochemical assessment of the content of this antigen would increase the value of the work. Our focus for the future is to perform an analysis on a more representative group of patients, as well as a more detailed diagnosis in the context of atherosclerosis and diabetes, which may affect the intensity of glycation in a group of patients with thyroid tumors.

Glycation products were accumulated in pathological thyroid tissue at a similar level to tissue surrounding the tumor. One cannot say that AGEs are concentrated in oncogenic tumor. The extracellular matrix certainly was an area in which the glycation process was the most intense. At least a part of the products of fluorescent AGEs correlates positively with glutathione, which proves the hypothesis that glycation stress leads to an increase of oxidative stress and to an increase of antioxidant mechanism intensity. Our study presented the occurrence of free-radical reactive nitrogen forms by proving nitric oxide synthase expression in thyroid tissue. The presence of AGE receptors, RAGE, as well as scavenger receptors type A and B were also detected. These proteins occur in endothelium, follicular cells, and sometimes in macrophages. SR-A expression is higher than SR-B expression in healthy as well as in pathological sections of the thyroid gland and in adipose tissue surrounding the tumor. It turns out that galectin-3 remains for now the only one of the AGE receptors thought to be a marker of thyroid diseases. RAGE, SR-A and SR-B are not expressed in a significantly bigger number in specific types of thyroid cancer. We did not observe a significant expression of these receptors in the centre of the tumor and in the surrounding tissue. We do not consider fluorescent AGEs as a marker of thyroid disease. Although the method of determination of these compounds is simple and inexpensive, it is unspecific [20,37]. The presented results, compared and verified in the literature, can have a practical clinical use. Current trends in medicine stress prevention and the treatment of metabolic diseases via antioxidant usage. Our paper refers to this trend. Patients are recommended to take antioxidants: vitamin C, E and selenium [38]. In endocrinology, not much attention has been paid so far to anti-glycation strategies. There are factors that can counteract the formation of AGEs or eliminate the effects of their formation. RAGE antagonists with properties that block oxidative stress arising from this receptor have also been known and classified [39]. In our opinion, at least in some groups of patients with thyroid pathology, an anti-glycative therapy should be supplemented. In the literature the first reports have recently appeared regarding the fact that scavengers of methylglyoxal and other glycation inducers merit investigation as potential therapeutic strategies for malignant thyroid cancer [6]. We conclude that in-depth disentanglement of the patho-mechanism of the creation of thyroid changes in terms of glycation and oxidative stress may turn out to be relevant for clinical treatment in surgical endocrinology.

## Figures and Tables

**Table 1:** Characteristics of the study group.

	Papillary Cancer *n* = 4	Squamous Cell Carcinoma *n* = 1	Follicular Adenoma *n* = 3	Nodular Goitre *n* = 29
Age (years) (Mean +/− SD)	65.8 (±5.4)	65	61.3 (±14.0)	53.0 (±14.0)
Gender (% of women)	100	100	66.7	82.76
TSH (µIU/mL) (Mean +/− SD)	1.09 (±0.52)	2.61	1.4 (±0.54)	1.05 (±0.5)
FT4 (pmol/L) (Mean +/− SD)	13.94 (±0.47)	14.4	12.30 (±4.82)	14.53 (±1.86)
FT3 (pmol/L) (Mean +/− SD)	4.51 (±0.93)	4.59	5.61	6.36 (±0.75)
Haemoglobin (g/dL) (Mean +/− SD)	13.93 (±0.61)	13.3	13.97 (±0.91)	13.65 (±1.13)
Fasting glucose (mg/dL) (Mean +/− SD)	107.5 (±20.5)	112	97	94.4 (±11.06)
Creatinine (mg/dL) (Mean +/− SD)	0.72 (±0.05)	0.88	0.77 (±0.05)	0.70 (±0.12)
MAGE (µg/mL) (Mean +/− SD)	1069.0 (±628.2)	1704.5	1000.9 (±275.7)	1017.6 (±462.7)
Fluorescent AGEs (AU) (Mean +/− SD)	439.6 (±75.9)	311.2	339.0 (±25.2)	658.8 (±408.6)
Pentosidine (AU) (Mean +/− SD)	413.0 (±91.3)	550.3	559.1 (±371.2)	612.5 (±232.9)
Compounds with thiol groups (mM) (Mean +/− SD)	0.45 (±0.32)	1.36	0.59 (±0.37)	0.59 (±0.37)
MDA (nM) (Mean +/− SD)	0.85 (±0.09)	1.35	0.96 (±0.24)	0.94 (±0.27)

**Table 2:** The results of correlation analysis between analysed parameters—the first number shows correlation coefficient *r*, the number in brackets shows *p*.

*r*	TSH (µIU/mL)	FT4 (pmol/L)	HGB (g/dL)	Creatinine (mg/dL)	MAGE (µg/mL)	Fluorescent AGE (AU)	Pentosidine (AU)	Tiol Groups (mM)	MDA (nm/L)	Age (Years)
TSH [µIU/mL]	1.000									
FT4 [pmol/L]	−0.395 (0.046) *	1.000								
HGB [g/dL]	−0.262 (0.178)	0.172 (0.402)	1.000							
creatinine [mg/dL]	0.369 (0.070)	−0.286 (0.176)	0.276 (0.163)	1.000						
MAGE [µg/mL]	−0.037 (0.852)	0.121 (0.555)	0.035 (0.856)	0.266 (0.181)	1.000					
Fluorescent AGE [AU]	−0.071 (0.718)	0.079 (0.702)	−0.003 (0.988)	0.352 (0.071)	−0.168 (0.320)	1.000				
pentosidine [AU]	0.096 (0.626)	−0.275 (0.174)	0.118 (0.534)	0.158 (0.432)	0.129 (0.448)	0.137 (0.420)	1.000			
tiol groups [mM]	0.335 (0.082)	−0.025 (0.904)	−0.099 (0.602)	0.278 (0.160)	0.134 (0.429)	0.361 (0.028) *	−0.139 (0.411)	1.000		
MDA [nm/L]	−0.011 (0.956)	0.234 (0.251)	0.005 (0.980)	−0.122 (0.544)	0.137 (0.425)	−0.017 (0.921)	0.216 (0.205)	0.068 (0.694)	1.000	
Age [years]	0.086 (0.664)	−0.427 (0.030) *	0.071 (0.709)	0.020 (0.922)	−0.141 (0.419)	−0.040 (0.819)	0.174 (0.317)	−0.049 (0.780)	−0.081 (0.649)	1.000

* *r* < 0.05. TSH- Thyroid Stimulating Hormone; FT4- Free Thyroxine; HGB- HaemoGloBin; MAGE- Melibiose-Derived Advanced-Glycation End-product; Fluorescent AGE- Fluorescent Advanced-Glycation End-product; MDA- MalonDiAldehyde.

## Data Availability

Data availability from the authors for request.

## Abbreviations

Abs	Absorbance
AGEs	Advanced Glycation End-products
ATC	Anaplastic Thyroid Carcinoma
BSA	Bovine Serum Albumin
ATP	Adenosine TriPhosphate
CD204	Cluster of Differentiation 204
CD36	Cluster of Differentiation 36
CEL	*N*(ε)-CarboxyEthylL*ysine*
CML	CarboxyMethylLysine
cNOS	constitutive Nitric Oxide Synthase
DAB	3.3′-DiAminoBenzidine
DM	Diabetes Mellitus
DNA	DeoxyRibonucleic Acid
DPX	Dibutylphthalate Polystyrene Xylene
DTNB	5.5′-DiThiobis-2-NitroBenzoic acid
ELISA	Enzyme-Linked Immuno-Sorbent Assay
eNOS	endothelial Nitric Oxide Synthase
FEEL-I	Link domain-containing scavenger receptor-I
FEEL-II	Link domain-containing scavenger receptor-II
FT3	Free Triiodothyronine
FT4	Free Thyroxine
FTC	Follicular Thyroid Carcinoma
Gal-3	Galectin-3
GSH	Glutathione
GR	Glutathione Reductase
GSHPx	Glutathione Peroxidase
HbA1C	Glycosylated Hemoglobin type A1C
HDL	High-Density Lipoprotein
HGB	HaemoGloBin
HMW-AGE	High Molecular Weight-Advanced Glycation End products
HRP	HorseRadish Peroxidase
IgE	Immunoglobulin E
IgG	Immonoglobulin G
LDL	Low-Density Lipoprotein
LMW-AGE	Low Molecular Weight-Advanced Glycation End-products
LOX-1	Lectin-like OXidized low-density lipoprotein receptor-1
MAGE	Melibiose-Derived Advanced-Glycation End-product
MDA	MalonDiAldehyde
MOLD	MethylglyOxal Lysine Dimer
MSR1	Macrophage Scavenger Receptor 1
MTC	Medullary Thyroid Carcinoma
NADPH	Nicotinamide Adenine Dinucleotide Phosphate reduced
NO	Nitric Oxide
NOS-3	Nitric Oxide Synthase-3
OPD	O-Phenylene Diamine hydrochloride
PBS	Phosphate Buffered Saline
PBST	Phosphate Buffered Saline with Tween-20
PTC	Papillary Thyroid Carcinoma
RAGE	Receptors for Advanced Glycation End-products
RNS	Reactive Nitrogen Species
ROS	Reactive Oxygen Species
SCTC	Squamous-Cell Thyroid Carcinoma
SCARA1	Scavenger receptor class A member 1 gene
SCARB1	Scavenger receptor class B member 1 gene
SDS	Sodium Dodecyl Sulfate
SR	Scavenger Receptors
SR-A/SRA-I	Scavenger receptor class A member 1
SR-B/SRB-I	Scavenger receptor class B member 1
TBA	ThioBarbituric Acid
TCA	TriChloroacetic Acid
T3	Triiodothyronine (L-3,5,3′-Triiodothyronine)
T4	Thyroxine (L-3,5,3′,5′-Tetraiodothyronine)
TAMs	Tumor-Associated Macrophages
TSH	Thyroid Stimulating Hormone
WHO	The World Health Organization
